# To Teach or Not to Teach: An International Study of Language Teachers’ Experiences of Online Teaching During the COVID-19 Pandemic

**DOI:** 10.1007/s42979-022-01323-6

**Published:** 2022-08-04

**Authors:** Ching Ting Tany Kwee

**Affiliations:** School of Education, Faculty of Arts, Design and Architecture, The University of New South Wales, Sydney, Australia

**Keywords:** Online learning, Language teacher, Social Cognitive Career Theory, Interpretative Phenomenological Analysis

## Abstract

Schools have been switching to online learning to ensure students’ learning continuity during the COVID-19 pandemic. However, there is a paucity of studies examining language teachers’ motivations and decisions for continuing online teaching in the future. This study aims at investigating the significant factors influencing language teachers’ motivations and decisions on online teaching. Based on the aim of this study, three research questions guided this study: (1) What was language teachers’ experience of online teaching? (2) What motivates language teachers to teach online after the COVID-19 pandemic? (3) What demotivates language teachers to teach online after the COVID-19 pandemic? Eight language teachers coming from six countries and regions, namely, Australia, Canada, Hong Kong, New Zealand, Russia, and Taiwan, were selected to have two one-on-one semi-structured interviews. The researcher used Social Cognitive Career Theory as a theoretical framework and Interpretative Phenomenological Analysis as the methodology to examine language teachers’ experiences in-depth. This study found that better time management and a positive learning environment are the reasons for continuing online language teaching, while personal beliefs on education and negative teaching outcome expectations are the reasons for stopping online language teaching. The findings can provide insights for the education institutions, school management and policy-makers to devise appropriate strategies to boost language teachers’ motivations to incorporate online teaching in the post-pandemic era.

## Introduction

### Background of the Study

COVID-19 pandemic has brought drastic change to the learning strategies and environment to K-12 students and teachers [1, 2]. During the COVID-19 pandemic, total and partial school closure is a popular strategy adopted by various countries to control the spread of the virus. Countries and regions like India, Hong Kong, China, Indonesia, Australia, and Algeria are the case at a point. According to the United Nations’ report [2], approximately 1.58 billion students had to transit to remote learning during the pandemic. One instant benefit brought by such a transition is ensuring students’ learning continuity. Teachers use various video-conferencing tools such as Google Meet, Zoom and Microsoft Teams for live sessions [3]. They use Google Classroom for homework submission and check students’ daily work. They also use some online platforms, learning tools or other add-on functions developed by private companies to facilitate their online teaching, such as collaborative blackboard [4]. With both the advantages and disadvantages of online learning, a lot of discussion centres on whether online teaching and learning can become a ‘new normal’ in the post-pandemic era [1, 3].

### Purpose of the Study

This study aims at investigating the significant factors influencing language teachers’ motivation for online teaching in the post-pandemic era. By examining language teachers’ online teaching experiences during the COVID-19 pandemic internationally, the researcher identified personal and contextual factors influencing their decision of online teaching. It not only serves as the predictors of their future choices of the medium of delivery, but also fills the research gap on the paucity of research on language teachers’ online teaching experiences, motivations and decisions.

Based on the purpose of the study, this study was guided by three research questions:

1. What was language teachers’ experience of online teaching?
2. What motivates language teachers to teach online after the COVID-19 pandemic?
3. What demotivates language teachers to teach online after the COVID-19 pandemic?

## Literature Review

This section aims to position this research among the current studies by accentuating what the scholars have previously done and identifying the research gaps in their study. Research on online learning and teaching has been done to examine the impacts, benefits and drawbacks of online or distant learning [1, 5, 6].

**Fig. 1:**
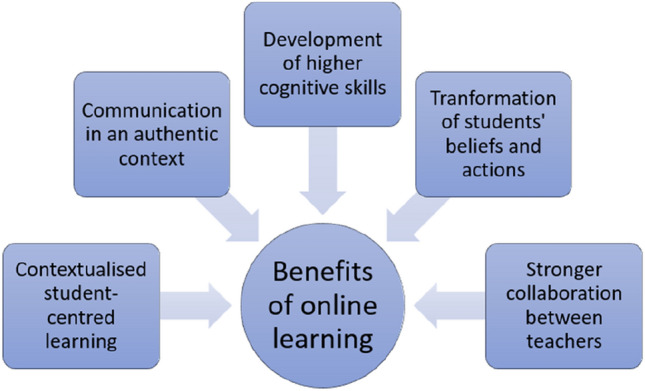
Benefits of online learning for students and teachers

With the mastery of technological skills and familiarity with various software and online learning platforms, teachers enjoy the benefits of effective teaching and learning [3, 6]. Many scholars [5–8] believed that online learning is beneficial to students’ learning due to its interactive features. Such interactive features can make online teaching more student-centred, whereby teachers can design more interactive tasks to facilitate learning [6, 8]. For example, teachers can utilise those ‘down-to-earth’ features like social media, forums and chats to contextualise their teaching and engage their students in an active and frequent discussion [5, 9]. Since such interaction and communication are conducted in an authentic context, the language used can be related to their real lives, and thus, students can then express meaning without pressure. For example, the informal and colloquial expressions students use can ease their pressure of learning, leading to a more open discussion and exchange of thoughts [5, 10]. Since language learning always needs communication and interaction, interactive tasks on the online learning platforms allow teachers to give timely feedback [9, 11]. Students have ample opportunities to move from information delivery to different ways of processing information, which need higher cognitive skills like critical thinking. Students can then challenge each other in their valued discourses and bring reflection and understanding on values, beliefs and cultures [7, 8]. Such reflection can later transform beliefs and actions, which align with the future trend of education [12, 13]. The transformative power of online teaching and learning lies not only in the benefits to ordinary school children. Such new mode of learning brings great accessibility of knowledge to those students from lower socio-economic background or living in remote or regional areas, whereby access to educational resources is scarce [4, 10, 14]. Some scholars [3, 6, 15] also pointed out that teachers perceive remote or online learning as an opportunity to foster more active collaboration between colleagues. For example, although teachers believe that the transition to the online teaching platform can be too rapid and bring some nuisance, they also believe optimistically that they can team up and share workload between colleagues. With these advantages, some teachers perceive online teaching and learning can be successful during the pandemic and possibly after the pandemic. Figure 1 summarises the benefits of online learning for students and teachers.

**Fig. 2:**
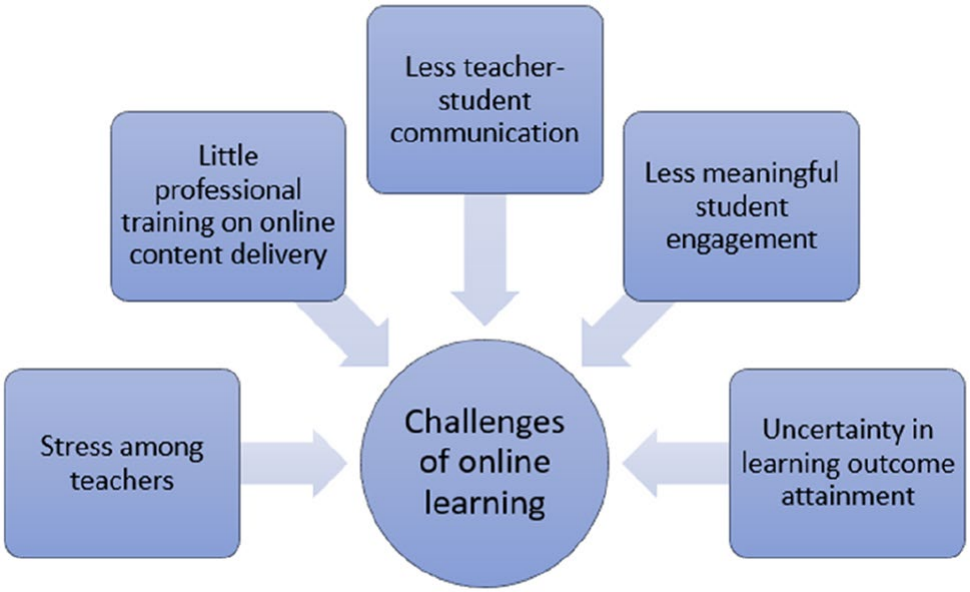
Challenges of online learning

Nevertheless, some scholars [1, 16, 17] held a more cautious or conservative view towards online teaching and learning. Although some studies suggested that less workload from teachers can possibly be attained during the implementation of online teaching, some scholars suggested otherwise. For example, some scholars [1, 16] suggested that such rapid and drastic transition from the traditional mode of learning to online one may induce stress among teachers. Teachers may have to spend extra time to prepare the materials, thereby leading them to become more vulnerable to burn-out and exhaustion [16]. Besides, some teachers may not be used to the technologies used in their online lessons. They have to spend extra time to adapt to both the technologies and new pedagogies for effective teaching [2, 16]. However, some teachers reflected that they have received little or almost no training on the online delivery mode, and it further deteriorates their mental well-being. Moreover, teachers are also concerned with the lesson delivery and learning outcomes. Although there is a substantial amount of course and subject contents being delivered online, it does not guarantee the same amount of communication time between teachers and students [6, 18]. For example, some students have reflected that in their online lessons, it has been more difficult for them to get feedback from teachers owing to the little communication time between them [6, 18]. From the teachers’ perspective, some teachers are worried that the physical absence of teachers and peers can lead to a less meaningful engagement of students in lessons [19, 20]. Due to the communicative nature of language, such possibility of a decrease in engagement may lead to some uncertainties in teaching effectiveness and students’ learning outcomes. As for students from lower socio-economic backgrounds, attending live lessons can be a challenge and their incapability of attending class and completing their assignment can further lead to the non-attainment of teaching goals [1, 2, 17]. Figure 2 summarises the challenges of online learning.

Previous studies [1, 3, 5, 6] have identified both advantages and disadvantages of implementing online learning by examining the learning outcomes and the effectiveness of various teaching methodologies alongside the backgrounds of teachers and students. However, very few studies [3, 16] examined teachers’ teaching experiences in relation to online teaching. For example, communication has been a crucial part in developing literacy and fluency, as well as the mastery of content and cognition skills. Very few studies examined how language teachers perceive the feasibility and mode of communication during their online teaching. One of the shortcomings of focussing on the effectiveness and outcomes is that the complex web of variables in rich and in-depth lived experiences are reduced to mere stimulus and behaviours [21, 22]. Successful teaching encompasses myriad personal and contextual factors, whereby these factors intertwine and influence teachers’ motivations and behaviours reciprocally [23, 24]. Such reduction from choices or decisions to behaviours may lead to a neglection of the sense-making process of the decisions, whereby the mental process of how and why language teachers make a decision of online teaching in relation to various personal and contextual variables such as their personal belief, education background, learning experiences, supports from school management and colleagues, which can be influential, may not be thematised for discussion [25, 26]. To shed light on the influential personal and contextual variables, it is important to look into the mental process of language teachers making decisions on online teaching.

## Theoretical Framework

Social Cognitive Career Theory (SCCT) was chosen as the theoretical framework for this study. SCCT is career-oriented developed specifically to examine how career decision is developed and persisted through examining the relationship between human capabilities, behaviours and environment [25, 26]. Unlike other career theories which perceive individuals as passive respondents to past or present environmental influences, SCCT affirms individuals as active agents who can bring changes in themselves and the environment in the process of career development.

There are three interlinked key constructs in the SCCT, namely, self-efficacy, outcome expectation, and performance goal attainment [25, 26]. Self-efficacy is defined as people’s beliefs about their capabilities to reach a specific performance. If individuals have successful past experiences, positive feedback from their actions, a favourable environment and greater satisfaction or well-being from their actions, they will form a set of self-affirming beliefs and become more confident in performing a similar task in future. Outcome expectations include three major forms: social, material, and self-evaluative anticipated outcomes and personal beliefs influencing one’s engagement in some particular behaviours. Performance goal attainment in SCCT includes meeting basic survival needs, achieving specific intrinsic or extrinsic ends and actualising one’s self-objectives, whereby comprising measures of success, mastery of skills, and perseverance at problem-solving. For individuals having a similar level of ability, those who have higher self-efficacy and more positive outcome expectations are more likely to establish higher performance goals and aim for more challenging attainments [25, 26]. With the key constructs of the SCCT, namely self-efficacy, outcome expectation and performance goal attainment, this study could examine the interplay between personal and contextual variables influencing the language teachers’ career decisions of continuing or stopping online teaching.

As mentioned in the previous section, teachers’ decisions of adopting online teaching are influenced by myriad personal and contextual variables [23, 24]. However, the decision-making process is yet to be known as there is a lack of holistic examination of these variables. The SCCT accentuates the interplay between individual, environmental, and behavioural variables to draw a more precise representation of the decision-making process of adopting online teaching, thereby the cognitive and psychological mechanism in relation to personal factors, social supports and barriers could be outlined [25, 26]. Second, with the key constructs of the SCCT, the researcher was able to identify the specific variables influencing language teachers’ motivation and confidence in online teaching in relation to how they postulate the attainment of the teaching outcomes [27, 28]. Thus, it allows the researcher to examine specific beliefs and pedagogical considerations in language learning and online teaching. Third, the SCCT accentuates its transformative and predictive power of self-efficacy, whereby an individual is an active agent having the capabilities to combat the unfavourable environment to attain one’s performance goals [29, 30]. By examining the language teachers’ teaching experiences with the SCCT lens, this study was able to identify the key personal and contextual variables influencing language teachers’ online teaching decisions, thereby allowing the possibility of devising intervention strategies to orient their choices in future [29, 31].

## Methodology

The researcher selected a qualitative study design to examine language teachers’ motivation and decisions. Unlike quantitative studies which aim at generating objective statements about language teachers’ self-efficacy and performance goal attainments, qualitative study design provides an advantage to explore the language teachers’ online teaching experiences [32–34]. To give a full account of ‘how’ and ‘why’ language teachers decide to continue or quit online teaching, the researcher adopted Interpretative Phenomenological Analysis (IPA) as the methodology of this study. IPA extends from the traditional Husserlian and Heideggerian phenomenology, describing and interpreting the participants’ experiences by co-constructing the participants’ experiences in their lifeworld alongside their sense-making process of such experiences [34–36]. Since teachers’ online teaching experiences are influenced by numerous intertwined personal and contextual factors, IPA values the participants’ own voices in expressing their own experiences, enabling the unfolding of both their lived stories and its sense-making without distortion. Together with the use of the SCCT key constructs, the researcher was able to go beyond the apparent content, unfold the reciprocal relationship between these variables and bring the hidden meaning of their online teaching experiences onto the surface. As a result, the study could obtain rich and in-depth data and a thorough understanding of how language teachers are motivated and make decisions on their future online teaching [25, 26, 36, 37].

### Participants

The IPA handbook [33, 34] was used as the guideline to select eight in-service language teachers for the study. IPA advocates a small sample size between three and ten participants to gain an in-depth understanding of the participants’ lived experiences. In this study, all these participants shared a similar lived experience of conducting online language teaching during the pandemic. To ensure the credibility and trustworthiness of this study, the researcher obtained a fairly homogenous sample of participants through purposive sampling [33, 34, 38]. The participants have to meet the following criteria:

- is currently teaching in a public or private school;
- has completed the initial teacher training;
- has been teaching a language subject;
- has conducted online teaching for at least 2 months during the COVID-19 pandemic.

Following the guidance of IPA handbook and suggestions by other IPA scholars [33, 34, 39, 40], the researcher carefully selected eight participants from six countries with different years of experience. First, including participants from different countries of origin allowed the researcher to investigate how contextual variables like curricula, pedagogies, classroom settings, technological knowledge and accessibility could influence language teachers’ motivation for online teaching [26, 34, 41]. Second, including language teachers with different years of experience could possibly probe into how various personal variables like personal beliefs, education background, previous learning and teaching experiences influence their self-efficacy and thus their motivation [26, 34, 41].

This study was conducted in compliance with the Helsinki Declaration, whereby the researcher endeavoured great effort to protect the human subjects participated in this study. Since all participants are in-service teachers, the researcher used pseudonyms to protect their identities from their current and further employers [42, 43]. Table 1 presents the participants’ demographic details.

**Table 1:** Demography of the Participants

Name	Gender	Age	Subject taught	Years of experience	Campus location
Melina	F	Mid-30s	English, Literature in English, English as an Additional Language/ Dialect	8	Australia
Matilda	F	Late-30s	Chinese Language and Literature, Chinese as Second Language	10 +	Australia
Sam	M	Early-40s	Chinese, Chinese Literature	10 +	Hong Kong
Anita	F	Mid-20s	Chinese, Chinese Literature	2	Taiwan
Catherine	F	Mid-20s	English, English Literature, English as Second Language	3	Canada
Ivan	M	Mid-30s	English, English as Second Language	5	Canada
Jason	M	Mid-40s	Chinese, Chinese as Second Language, English as Second Language	10 +	New Zealand
Natalie	F	Late-30s	English as Second Language	2	Russia

### Data Collection and Analysis

The researcher conducted two online one-on-one semi-structured interviews with the eight participants [44]. Before the interviews were conducted, the researcher sent a letter of consent to each participant to gain approval for conducting the research with the participants [42, 43].

One-on-one interviews allowed the participants to share their lived experiences without stress in a more comfortable private setting, thereby increasing the possibility of generating rich and in-depth data for the study [42, 45]. Semi-structured interviews allowed the researcher to ask open-ended and follow-up questions in relation to the participants’ concerns and interests for more in-depth discussion [33, 34]. In the semi-structured interviews, the researcher also invited them to share their lived stories in their own words under the guidance of the interview protocol. The researcher followed the guidelines recommended by the IPA handbook and developed the interview protocol with the SCCT constructs, personal variables (e.g., language, race/ethnicity, family and education) and contextual variables (e.g., barriers, supports, classroom management, technical support and learning environment) in relation to their teaching experiences [26, 33, 34, 41]. The first interview was to establish rapport and empathy with the participants by understanding their background, personal beliefs and previous learning and teaching experiences [33, 34, 44]. The second interview was about their current inline language teaching experiences in their countries. It focussed on their present lived experience, specifically what they actually do in the job, their relationship with different stakeholders, challenges and tensions they face while conducting online language teaching, alongside their motivations, decisions and actions of continuing online language teaching [33, 34, 44]. Please see the interview questions in Appendix 1.

All the interviews were recorded and transcribed. They were recorded in a separate electronic device and transcribed verbatim [42, 46]. Each interview lasted from 62 to 123 min and contributed to 69 to 117 pages of written transcripts. Table 2 presents the details of the duration, number of pages of written transcript and number of interview questions in each interview per participant.

**Table 2:** Themes and subthemes

		Themes and subthemes
5.1		Better Time Management: Reasons for Continuing Online Teaching
	5.1.1	Less Time on Lesson Preparation and Homework Marking
	5.1.2	Fulfilment of the Familial Duties
5.2		Positive Learning Environment: Reasons for Continuing Online Teaching
	5.2.1	Better Classroom Discipline
	5.2.2	Stronger Learning Motivation
5.3		Personal Beliefs on Education: Reasons for Stopping Online Teaching
	5.3.1	Incapability of Attaining Social Equity
	5.3.2	Poor Communication with Colleagues
5.4		Negative Teaching Outcome Expectation: Reasons for Stopping Online Teaching
	5.4.1	Difficulties in Ongoing Observation and Evaluation
	5.4.2	Difficulties in Implementing Summative Assessment

After transcription, the researcher sent the transcripts to the participants for member-checking and approval to further process the data information before data analysis, thereby enhancing the credibility of this study [42, 46]., The researcher employed triangulation, whereby observing the recorded lessons and teaching materials provided by the participants to further ensure the validity of the collected data [42, 46].

After data collection, the researcher employed a general inductive approach to analyse the collected data [47, 48]. Employing iterative cycles of data reduction, the researcher first reduced the chunks of data to first-level themes by the open-coding technique. After that, the researcher generated 18 themes after open-coding. However, the researcher noticed the massive first-level themes emerged, thereby employing axial coding to reduce the number of themes and subthemes for standard reporting. As a result, four themes and eight subthemes emerged.

## Results and Discussion

While reporting the findings of this study, the researcher followed the suggestions from scholars [33, 39, 40] to combine both textual descriptions of the language teachers’ online teaching experience and discussion with the SCCT constructs so as to provide a holistic view of how the evidence substantiates the claims. To answer the research questions, the researcher categorised four themes and eight subthemes. Table 3 summarises the themes and subthemes of this study.

**Table 3:** Duration, number of pages of written transcript and number of interview questions in each interview per participant

Participant	Interview duration in minutes (first interview)	Interview duration in minutes (second interview)	Number of pages of written transcript (first interview)	Number of pages of written transcript (second interview)	Number of questions asked (first interview)	Number of questions asked (second interview)
Melina	68	89	73	94	23	31
Matilda	91	95	100	101	28	27
Sam	78	123	84	117	27	34
Anita	93	69	101	73	33	25
Catherine	80	78	88	80	27	23
Ivan	62	88	69	93	21	28
Jason	102	93	112	96	30	29
Natalie	117	104	115	110	35	33
Mean	86.4	92.4	92.8	95.5	28.0	28.8
Median	85.5	91	94	95	27.5	28.5
Standard Deviation	18.1	16.3	17.1	14.4	4.7	3.8

### Better Time Management: Reasons for Continuing Online Teaching

Some researchers [3, 16] suggested that teachers’ well-being was severely affected due to the extra working hours spent on lesson preparation and training for the online delivery mode. This study reflects another view that language teachers felt that they can better manage their time as they can spare less time on preparation and homework marking. Alongside the benefits of working from time, they can balance their familial role and role as a teacher. As a result, they attain better well-being and have a stronger desire for continuing online teaching in future [25, 26].

#### Less Time on Lesson Preparation and Homework Marking

Previous studies [3, 16] suggested that teachers were bombarded by stress due to an increasing workload in lesson and assignment preparation, leading to substantial difficulties in time management. This study reflected that language teachers did not feel that time management is a critical issue due to their familiarity with different features in the online learning platforms.

*“I think I’m doing it well… It’s not something really demanding…Before the online lessons, I just have to upload the notes to Google Classroom. We don’t need to photocopy the handouts for the students. In the online lesson, I can instantly access their classroom and give feedback… It saves a lot of time. In Zoom, I just have to open the notes and annotate it…Once I switch off the camera, I send the annotated notes back to students… It’s fast.” (Melina, Australia)*

This study found that language teachers attain a sense of achievement when they can manage the online learning tools, whereby offering them specific teaching-relevant supports like homework submission management and effective feedback system. When they achieve a sense of professional competencies, they think positively about online learning and the associated work demand [19, 20]. Another participant shared a similar view

*“We need to give two types of feedback – one on their homework... I use Google Classroom to collect and return their homework…No hassle… I can check who has submitted the homework. Before I have to ask the subject representative to count and check. Now, it [Goggle] can generate a list instantly…I haven’t been that efficient during my 20 years of teaching career... I’m superb in marking students’ writing…” (Sam, Hong Kong)*

An increase in work efficiency fosters language teachers’ greater engagement in online teaching. Unlike some scholars [3, 16] suggesting that teachers’ working hours had been increased due to preparation of personalised assignments and feedbacks in a distant learning environment, participants in this study suggested that online teaching boosted their work efficacy by reducing their time spent on such preparation. Another participant said

*“I don’t agree that online teaching means having more work to do. We can manage… It’s just like marking their work on paper…but it saves time. I used Turnitin [an online grading and feedback system]. I saved some marking codes for frequently used comments… While marking their work, I just highlight… drag and drop… add comments… and specific suggestions for improvement for that particular section of their work.” (Matilda, Australia)*

Unlike previous studies [3, 6] suggested that teachers have to spend extra time to prepare a rapid conversion to online teaching, this study found out that language teachers did not find such transition too abrupt as they were already fairly familiar with the technological requirements of various online teaching tools and platforms. Such familiarity makes them spend less time on chores and daily routines, thereby attaining happiness and job satisfaction while meeting the job demands [19, 20]. This affirms with the SCCT hypothesis that such positive affection can be an extra source of self-efficacy with a self-aiding effect on postulating a scenario of having similar success and positive affection in future, thereby increasing the motivation and affirming the decision of continuing online language teaching in future [26, 41].

#### Fulfilment of the Familial Duties

Some scholars [3, 19, 49] suggested that online teaching influences teachers’ personal life greatly as they have to work for longer hours and juggling between work and family. In this study, although language teachers have shown their concerns on their mental well-being, generally, they did not feel that they are facing problems like burn-out and exhaustion. Instead, they feel that online teaching gives them an opportunity to fulfil their duties as both a teacher and a parent. One participant said

*“I stay at home… He [the participant’s son] is also having online schooling… He is happy to see me all day round. I teach him how to play the guitar… We play. We cook…I take him to sleep…I feel like I’m Dad, a real one…It’s really ironic that as an educator you spend more time with others’ children than your own kids… I like my family… If possible, I want to keep this [online teaching].” (Jason, New Zealand)*

While working from home, language teachers can spend more time with their family, thereby fulfilling their familial duties as a parent. Such fulfilment reflects the attainment of their personal goals, which leads to better well-being as their family life is enriched. Such positive affection makes them appreciate the advantage of online teaching [26, 41]. Another participant shared a similar view

*“I really wish to do online [teaching] forever…I meant I don’t want the pandemic to continue…But it’s really good to stay at home to work… [Before the pandemic] I could only have little time for lunch… Work and travel took lots of time…I felt tired… I didn’t really wanna prepare dinner…I felt so sad when I couldn’t make it… Now… I have more time. I saved more than two hours in travelling each day. I can prepare brekkie, lunch and dinner… I can have lunch with my daughters… Hot meals… My daughters really like the noodles and spring rolls…I’m happy with this change.” (Melina, Australia)*

Participants in this study reflected that online teaching saved their time in travelling to work and such time could be used more effectively in forging a closer bond with their families. Language teachers agreed that such fulfilment of familial duties brings them better well-being due to an improvement of relationship and satisfaction of being a parent or a caretaker. Such work arrangement can reduce language teachers’ stress and harmonise their work and personal life [50, 51]. For example, another participant, Sam, also expressed that better time management due to working from home enriched his family life. He said

*“I can utilise my time better…Now I don’t have to travel to work and switch classrooms [between lessons]… I only have to work half day… It’s easier to arrange some time we ‘do something together’. We have been married for twenty years… Both of us are teachers… We didn’t really have time to ‘do things together’ with children. Usually it’s my parents or my wife’s parents take care of the kids… Sometimes I had meetings at school or extra lessons. Sometimes she had to attend those PTA [Parent-teacher Association] meetings… Now at least we had half-day free. We can just go down to the promenade for a walk. Happy family life, right?” (Sam, Hong Kong)*

Personal goals play a pivotal role for an individual to select a career or make a career choice, whereby an individual’s perceptions of outcomes are crucial. According to the SCCT hypothesis, language teachers attain their personal goal due to the fulfilment of familial duties, whereby bringing more positive affection, better mental well-being and greater sense of satisfaction [26, 41]. Such attainment becomes language teachers’ self-efficacy belief in reinforcing the benefits of online teaching, and hence, they are more likely to continue online teaching in future.

### Positive Learning Environment: Reasons for Continuing Online Teaching

Some scholars [4, 5] raised concern whether online learning can offer a favourable learning environment for students. This study reflected that better classroom discipline and greater learning motivation could be guaranteed due to various functions offered on the video-conferencing and online learning platforms. These foster language teachers’ professional identities and increase their positive affections, and thus, they are more willing to continue online teaching after the pandemic.

#### Better Classroom Discipline

Previous studies [18, 52] suggested that teachers are generally worried about students’ discipline issues as they may be involved in disruptive behaviours due to their incapability of paying attention and focussing on online lessons due to the physical absence of teachers and peers. This study, however, suggested that language teachers generally found that classroom discipline is better in their online classes when comparing with the physical lessons they had with students at schools. One participant said

*“Zoom has got… video recording function… Microsoft Teams has also got a video-recording function. Sometimes I reminded the students that the lessons would be recorded. They would be more alert. They know that somehow they are being watched, not always… but it is possible.” (Melina, Australia)*

This study reflected that the monitoring function of the online video-conferencing platform creates a more disciplined classroom, owning to the potential power of surveillance. Being monitored as a part of the learning community gives students a less stressful but somehow powerful reminder of the norms they have to follow [53]. Under such circumstance, students are generally better behaved and show greater respect to teachers. As a result, teachers are generally more satisfied with the online teaching experiences. One participant said

*“Some students really like interrupting others in the class, especially during class discussion. I asked my students to switch on their webcams. They can sit still and sit properly… This is the respect they should pay… I feel they become more respectful to the teachers now. They [Students] know what they have to do. I don’t have to yell at them to ask them not to do this or that.” (Sam, Hong Kong)*

The instant video and audio functions influence language teachers’ self-efficacy positively as it increases their positive affection due to the respect paid by their students [26, 41]. Moreover, these platforms also grant teachers with the rights as the host (i.e., the language teacher), whereby having greater control of how the online meeting (i.e., the online class) can be conducted. Such control leads to an avoidance of disruptive behaviours, fostering better classroom discipline [54]. Another participant reinforced such idea

*“Now when they [the students] are reminded that the lessons are recorded, they know they have to follow the class rules. With the mute function, only the one presenting or sharing ideas talks. Other have to mute themselves to avoid background noise. As teachers, we can better avoid unwanted behaviours. I feel I’m really teaching and I’m happy to have more online lessons in future.” (Ivan, Canada)*

Some other functions offered by these platforms provide preventive measures for teachers to foster better classroom discipline, thereby reinforcing teachers’ professional identity as students pay more respect to the teachers and their teaching [26, 55]. Another participant said

*“I can focus on my teaching. I can finish my teaching contents in Zoom. While having lessons at school, students like chipping in and asking silly off-track questions. In Zoom I managed the class better. While I am giving instructions, I can mute all the participants… I unmuted all… during discussion. Everyone can listen in a quiet environment. Seems I can teach more than ordinary school days.” (Natasha, Russia)*

Language teachers are able to attain greater positive affection and satisfaction which engrave their professional identity during online teaching. According to the SCCT, such positivity, alongside the attainment of their teaching goals, boosts the language teachers’ self-efficacy. As a result, they are more likely to postulate a similar success while conducting online language learning in future, thereby having a stronger desire for continuing their online teaching in the post-pandemic era [26, 41].

#### Stronger Learning Motivation

Previous studies [5, 39] suggested that online learning can arouse students’ learning motivation through interactive activities and visual and audio interfaces. This study also found that students’ motivation for language learning is boosted due to various features provided by the video-conferencing tools and online learning platforms, enhancing students’ language learning experiences as a more communicative one. One participant said

*“Before the pandemic, students didn’t like having reading classes. They didn’t want to read aloud. They are afraid of being judged… mispronunciation, misinterpretation… We did it in a way how questions appear in textbooks. Now I put more mini-discussions in the lessons… It is not just I ask a question and they answer, and then another question. They feel more free to share and talk… They asked each other questions: how, why, what… wh-questions… Students like it…They volunteered some great ideas. I’m so proud of them.” (Natalie, Russia)*

Participants in this study affirmed that technologies provide them greater support in facilitating teacher–student and student–student communication, whereby such communication is fostered effectively. Such communication is not just information exchange or simple IRF (Initiation-Response-Follow-up)/IRE (Initiation-Response-Evaluation), whereby patterns of interactions are confined [56, 57]. It helps to build a community of higher cognitive inquiries. Interaction is one of the important elements in an educational experience. Technologies provide the possibilities of more in-depth communication, whereby students utilise their higher order thinking skills such as critical thinking and system thinking to discuss and collaborate with their peers without pressure [54, 58]. Another participant said

*“My students used to be very shy to express their thoughts and opinions. It was always silent in lessons. They felt very strange to ask questions. Now in Zoom they can use the icons give reactions and responses… like raising hand… They are more willing to elaborate, give evidence or even challenge others’ views. It gives others different perspectives to analyse a text or think over a particular issue critically. I can hear their voices now... I’m proud of their achievement.” (Anita, Taiwan)*

Most participants suggested that online learning platforms provide a more comfortable and stress-free environment for students to respond to provocative and inferential questions, whereby students need to utilise their organisational and critical thinking skills. Positive affection for the attainment of teaching goals arises among language teachers, thereby boosting their job satisfaction and motivation in continuing their online teaching [26, 41]. Moreover, this study also found that some other features in online learning platforms further boost students’ motivation in learning, whereby enhancing language teachers’ confidence in online teaching

*“ClassIn [An online learning platform] allows students to collaborate to write a text. They can put their ideas of description, pictures… the stimulus and sample paragraphs… using sensory imagery there… I awarded each student a trophy [a feature in ClassIn app] when they completed a task… They liked the animation and sound effects…They asked for more.” (Melina, Australia)*

This study reflected that in-app features enable students to engage in lessons more actively through positive reinforcement as such positive reinforcement and reward mechanism boost students’ confidence in learning. Such experiences serve as the students’ background learning experiences, making them perceive and postulate similar success in their future learning [26, 41]. When language teachers are able to maintain students’ interest in learning, it is another indicator of successful online teaching [55, 59]. According to the SCCT, such performance goal attainment boosts language teachers’ self-efficacy. As a result, they are more motivated and more willing to continue their online language learning in future [26, 41].

### Personal Beliefs on Education: Reasons for Stopping Online Teaching

This study used the SCCT and IPA to permit insights into how personal factors influenced language teachers’ online teaching decisions. Being novel in the field, this study suggested that language teachers’ personal beliefs on education, particularly on social equity and collegial collaboration, negatively influenced their motivation for continuing online teaching due to the non-attainment of professional goals [26, 41].

#### Incapability of Attaining Social Equity

Scholars [1, 17] suggested that students from a lower socio-economic background are more vulnerable during online and remote learning due to their difficulties in accessing stable network and Internet-connected devices. This study affirmed such findings and found that such disadvantage challenges language teachers’ belief in attaining social equity via quality education. One participant said

*“People always think that everyone can get speedy internet access and an ipad… or just go online with mobile phone. But not every student kid. Some of my students have to share a device with their brothers and sisters. That’s why I can’t meet them sometimes. But their attendance counts for their CA (continuous assessment) marks. It means certain percentage of the marks is gone even before tests and exams. It’s unfair. No one helps them. My students feel powerless. I feel powerless too.” (Sam, Hong Kong)*

This study suggested that the prevalent beliefs in the society on equal access to Internet resources form a false perception that widens the learning gaps among school children and reflects in their unsatisfactory attendance and school results. Such non-attainment of learning outcomes makes language teachers cast doubt on their professional identity, whereby their belief in the education of achieving social justice is challenged [1, 60]. They feel that online teaching deprives disadvantaged children of opportunities to become successful, which contrasts with their belief in education. Another participant said

*“Every child is gifted… Every child deserves an opportunity to learn… That’s what I believe. But I’m not sure now if it’s doing the right thing. I feel like I’m widening the gap. I wanna be a teacher as I want a better world for the children. Now I’m the one widening the gap. We don’t always have stable internet connection. Even in Greater Sydney some areas don’t really have broadband internet. Sometimes the internet speed is affected by heavy rain and hailstorm… Sometimes they have to switch off their camera or video functions to ensure things won’t be too glitchy.” (Matilda, Australia)*

Unlike previous studies [4, 10, 14] suggesting that online teaching and learning helps to achieve social equity among those students who have limited access to educational resources and mobility, this study found that negative affection such as frustration arise among language teachers as they feel they are creating greater social inequality [1, 60]. This undermines their professional identity and further intensifies their feeling of incapability to attain their professional goal of pertaining a better future for their students [55, 61]. For example, another participant, Melina, agreed that such disadvantage is taken for granted in developed countries, leading to social inequality. She said,

*“Public schools do not really give a lot of supports to students. They just asked us to be understanding if students got disconnected during the lessons or they can’t attend the online lessons. Or they encourage us to put videos and learning materials online so students just need to access to the Google Classroom some time in a day. Those private schools got even online assemblies or morning workout periods. Students can use collaborative blackboard and discuss in breakout rooms. There is no doubt that private school students have more resources and advantages over the public school students. I’m sorry to say online learning is putting this fact onto surface.” (Melina, Australia)*

This study suggested that when there is a conflict between language teachers’ personal belief on quality education and the reality of implementing online learning, and they feel that they are unable to fulfil their professional goal as social equity cannot be achieved. According to the SCCT, such incapability, alongside negative affections, negatively influences language teachers’ outcome expectations and thus forming a self-hindering effect on the decision of continuing online teaching in future [26, 41].

#### Poor Communication with Colleagues

Previous studies [3, 16, 62] pointed out teachers’ concerns on ineffective communication with their colleagues during online teaching, particularly on collaboration on the preparation of teaching materials. This study explored how and why such concern can lead to language teachers not wanting to continue online teaching. Language teachers believed that poor communication with their colleagues negatively impacted their professional identity. They cast doubt on whether they can gain and provide collegial support, which they perceive as an essential part of their teacher identity [19, 20]. One participant said

*“According to the Australian professional Standards for Teachers, we have to engage with colleagues and improve practice. It’s really hard to seek and receive feedback these days. Each email takes a day or two for the replies… Sometimes the same email popped up in the inbox after several days… It’s kinda messy… It’s just one line or two when we’re in the staffroom.” (Matilda, Australia)*

When language teachers are unable to fulfil their performance goal to communicate effectively with their colleagues, they feel that they are not engaging in any professional dialogue and thus, they cannot fulfil the responsibilities and duties as a teacher, particularly when they do not receive adequate support [19, 20]. Such non-attainment of performance goals leads to frustration, which later turns into hesitation of continuing online teaching in future. Another participant reflected

*“Although there are lots of online communication tools like texts, emails and video calls, they are not as good as face-to-face talks... All our emails are about administrative arrangement or schedules of meetings... I missed the conversations in the staffroom…. I feel upset… We used to have show each other how to adapt some teaching materials or discuss our interpretation and analysis of works from a particular author… literature is always up to readers’ interpretations… We need such dialogue to construct our knowledge and understanding together.” (Catherine, Canada)*

This study reflected that language teachers’ frustration on the non-attainment of their professional goals can be related to their views towards the subject and language itself. Language teachers hold a constructivist view towards knowledge and a communicative view towards language, whereby they believe online teaching somehow hampers their chances of knowledge construction, particularly having communication on such construction [57, 63]. Such non-attainment collides with their personal beliefs on language and teaching and thus leading to doubts towards the effectiveness of online teaching. Another participant also shared a similar view. She said

“*I think we should be helping each other out to overcome the challenges… We were instilled with such belief since our initial teacher training… But it’s not the same as I thought… It’s frustrating…I didn’t really have chance to discuss with my colleagues whether a particular assessment is good for online learning... We didn’t really share our teaching materials as before. Sometimes I want advice… I felt overwhelmed… They [The participant’s colleagues] were too busy… They are just copying the email to each other. I don’t want this [online teaching and learning] to continue anymore.” (Natalie, Russia)*

This study found that language teachers felt overwhelmed when the reality of online teaching collided with their personal belief on being a successful teacher, whereby effective and meaningful communication between colleagues is one of the successful criteria [16, 64]. The non-attainment of such professional standards is detrimental not only to their professional identity, but also to their self-efficacy. Specifically, according to the SCCT, language teachers’ self-efficacy decreases due to a denial of their ability in attaining such performance goal, leading to the decision of termination of online language teaching in future [26, 41].

### Negative Teaching Outcome Expectation: Reasons for Stopping Online Teaching

Students’ assessments come in the forms of assessing their engagement in activities and reviewing their work as both process and product [65, 66]. Although previous studies [5, 10] suggested that teachers are able to assess students’ engagement on the online platforms, this study reflected that language teachers felt challenging in both giving ongoing evaluation of students’ performance and executing tests and examinations. The postulation of negative teaching outcomes diminishes their motivation for continuing online teaching in future.

#### Difficulties in Ongoing Observation and Evaluation

The study showed that language teachers feel positively towards the different kinds of feedback and rewards they can offer to their students to facilitate their learning. Although previous studies [5, 10] suggested that there are features like discussion forums to access students’ learning progressions, however, language teachers are very cautious and not very confident due to the uncertainty of ongoing observation and evaluation of students’ learning outcomes. One participant said

*“Sometimes teaching is like a monologue…They all muted and I am the only one talking… Teaching is done in one-way… I have covered all the contents…When I deliver the content well, I have the illusion that I teach well... I have to admit that even I asked all my students to switch on the cameras, it’s still very hard for me to observe each of their facial expressions…whether they are confused or not… In fact, it is hard to know how much students have learnt.” (Sam, Hong Kong)*

Participants in this study reflected that even the instant video and audio functions on the video-conferencing platforms or other learning platforms may not be very effective in accessing students’ instant learning outcomes. This leads to the unfulfillment of the purpose of formative assessing, which is providing ongoing feedback with constant evaluation of their learning outcomes [67–69]. Such unfulfillment can have a self-hindering effect and leads to a negative postulation of future online language learning. Another participant echoed this view. She said

*“Before [the pandemic], I walked around and checked to see if they understand some concepts… I had some learning stations set in different corners in the classroom. I could observe how each of them [the participants’ students] worked. They could come and ask me if they didn’t know what to do…Now I feel insecure because I can never really tell how well they take everything in… I couldn’t imagine this happening for a few months or even a year… I might prefer going back to face-to-face teaching… With more interactions, I know how they learn. I feel more secure and confident.” (Catherine, Canada)*

When language teachers are unable to develop knowledge on how far students have reached, they are overwhelmed by a sense of insecurity and perceive that as a failing experience damaging their professional identity [26, 41]. To stop further destruction of their professional identity, language teachers tend not to continue online teaching in future. One participant said

*“When there are great discussions, you can see lots of language skills and higher cognitive skills are developing… Things are not always plain sailing… On some bad days… I meant really bad… It could be dead silence for the whole lesson… They did show up. They did later hand in their homework. But in the lesson it’s really scary. You didn’t know what they feel and think… I don’t want to guess what’s in their mind, what they really know or what they really learn…. I like the old way [face-to-face teaching].” (Anita, Taiwan)*

This study concluded that language teachers are quite sceptical towards students’ learning outcomes when active discussion did not happen in their online lessons. Consequently, they felt very difficult to evaluate their students’ learning outcomes instantly [67–69]. Such doubt deteriorates their confidence in teaching successfully at that moment and in future. From the SCCT lens, such failing experiences and uncertainty creates self-doubt and constitute a negative background experience that diminishes their self-efficacy. As a result, language teachers tend to avoid similar tasks in future, which is continuing online language teaching [26, 41].

#### Difficulties in Implementing Summative Assessment

The purpose of summative assessments is to measures students’ learning outcomes and achievements by comparing them to the pre-set standard or benchmark [64, 70]. However, language teachers in this study reflected that it is very difficult to achieve the purpose of such kind of assessment when teaching is conducted online. They raised concerns about the forms of assessments they can possibly conduct online. One said

*“I like the discussion and other functions like emojis and collaborative blackboard. But…learning is less effective when it’s something related to assessment… Some tasks… requires memorisation… and routine practice…like dictations… It’s hard to manage… I know in some countries teachers can ask students to set cameras to show their [students’] desks and half body… But we can’t…You can’t make sure students aren’t cheating.” (Catherine, Canada)*

Unlike previous studies [64, 71] suggested that the security measures like locking down web browsers, text comparison or plagiarism check tools can ensure fairness during examination and tests, this study reflected that language teachers are generally worried about the security and fairness issues when it comes to conducting summative assessment online. Participants in this study pointed out that having summative assessments on online platforms can be a challenge as students’ integrity and honesty are hard to guarantee. Another participant said

*‘It’s hard to do the half-yearly online. You don’t know whether they rely on some external support to complete their assessment… Some students can be really cheeky and they asked their tutors or language coaches to be ready by their side. They [students] sent them [the students’ tutors] the test paper. Some students asked their peers who had the assessment a period earlier to shot photos of the questions. Then they could finish it within half an hour. But somehow you don’t have evidence to report these cases...Though there is a declaration form, it doesn’t mean anything. It’s an honour system anyways.” (Melina, Australia)*

Many participants in this study reflected that they feel powerless in assessing their students in a summative manner. Such powerlessness stems from the incapability in attaining fairness, which is a crucial criterion of a well-designed assessment [72, 73]. When language teachers cast doubts on whether they can meet such criterion, it turns to an erosion of language teachers’ beliefs and values. Another participant shared such view. She said

*“I don’t feel we’re doing well on that [assessing students’ learning outcome]… We took away the essays in the trial examination. We can’t make sure students are copying their essays or memorise a prepared essay… Students only have to do unseen reading and writing. We can’t assess all the modules before HSC [Higher School Certificate examination]. Although they already have their term assessment of those modules, trial is another thing… They have to do it in a timed condition.” (Matilda, Australia)*

Apart from powerlessness stemming from the incapability of guaranteeing fairness, language teachers also feel frustrated when the power of assessment diminishes [72, 73]. Such negative affection reflects that language teachers are experiencing self-doubt, thereby postulating a negative professional image due to such non-attainment. According to the SCCT, both the negative affection and the non-attainment of professional goals lower language teachers’ self-efficacy as they perceive online teaching as a threat to the fairness and quality of summative assessment. As a result, they are less likely to continue online teaching in future [26, 41].

## Conclusion: Limitations and Future Research Direction

This study identified the personal and contextual factors influencing language teachers’ decision on adopting online teaching; nevertheless, this study shows several limitations. First, most of the participants come from developed countries and regions. Although some of them has raised the concerns on social equity and resources issue, their relatively higher socio-economic background may have influenced their perception of online teaching. Therefore, this study suggests that further research can be done later on teachers from developing countries or lower socio-economic backgrounds to understand better the influences of the proximal contextual variables on the supports and barriers on online teaching and learning. Comparative studies can be done across the higher and lower socio-economic backgrounds to better understand the correlation between the perception of social equity and online language teaching [26, 61]. Second, this study focuses on the online teaching experiences of language teachers. Some personal and contextual factors identified in this study are not directly related to language teaching and learning. This study suggests that further research can be done with teachers teaching other subjects such as Science, Technology Engineering and Mathematics (STEM), whereby their teaching experiences can be examined and compared with the language teachers’ ones. Thus, it can be better known whether some variables identified in this study are universal or subject-based [26, 41].

Despite the limitations, this study is the first international study examining the language teachers’ online teaching decisions by adopting an SCCT lens. This study has contributed to the current field of studies on online teaching and learning in three aspects. First, methodologically, this study is novel in examining language teachers’ online teaching experience through using IPA. Although there are studies studying teachers’ online experiences, they do not specifically study language teachers’ decision-making mechanism. Second, this study has identified both pushing and pulling factors influencing language teachers’ online teaching decisions. Such insights permit universities and other educational institutions to develop relevant professional development or learning courses to boost the motivation of language teachers to adopt online learning in the future. The findings can also provide insights for the education policy-makers and school management to review the current online learning policies and implementation, probing into the possibilities of implementing online teaching as a standard component in the curriculum in future.

## Appendix A Interview Questions

Interview One.

1. Can you tell me a bit about yourself? Please include background information about the country of your origin, race/ethnicity, gender, family, educational qualification, etc.
2. How would you describe your teaching position? Please include where you are working, your students, years of teaching in this school, mode of work, subject taught, duties, etc.
3. Before the COVID-19 pandemic, how did you usually teach language? How did students usually learn the language?
4. How do you think of the effectiveness of teaching and learning language in the way you have just mentioned?
5. What do you think is the most important element in language teaching and learning? Would you share more?
6. What do you think is the biggest hurdle in language teaching and learning? Would you share more?
7. Were there any opportunities for teachers to collaborate before the pandemic? Would you please share more?
8. Before the pandemic, did you have any experience in online language teaching? Would you mind sharing more?
9. Before the pandemic, how did you think about the benefits of online language teaching and learning?
10. Before the pandemic, how did you think about the drawbacks of online language teaching and learning?
11. Before the pandemic, how did you think about the effectiveness of online language teaching and learning?
12. Before the pandemic, have you ever considered conducting online language teaching? Why/why not?
13. Were there any professional activities on online teaching before the pandemic? Would you please share more?
14. Is there anything else you would like to share that you didn’t have a chance to speak about?

Interview Two.

1. How did you come to the decision to conduct online teaching during the COVID-19 pandemic?
2. How long have you been involved in online language teaching? Would you please share more about your experience?
3. What are the most positive factors enabling you to conduct online language teaching successfully?
4. What are the most negative factors hampering you to conduct online language teaching successfully?
5. Did you receive any support when you first start online language teaching during the pandemic? Would you please share more?
6. Did you face any barriers when you first start online language teaching during the pandemic? Would you please share more?
7. How did you keep yourself motivated while teaching online?
8. Do you think you have achieved your teaching goals? Would you please share more?
9. Compared to your view on online teaching before the pandemic, do you think your view has changed? Would you please share more?
10. Compared to your teaching before the pandemic, do you think it is easier or more difficult to obtain success? Would you please share more?
11. How do you feel about all this effort you are making to online language teaching?
12. How confident are you now about conducting online language teaching? Why?
13. What goals do you set for yourself if you have to conduct online teaching in the future?
14. Do you want to continue online language teaching after the pandemic? Why/why not?
15. Is there anything else you would like to share that you didn’t have a chance to speak about?
